# Application of radiomics-based prediction model to predict preoperative lymph node metastasis in prostate cancer: a systematic review and meta-analysis

**DOI:** 10.3389/fonc.2025.1577794

**Published:** 2025-06-20

**Authors:** Yanghuang Zheng, Yuelin Du, Biao Zhang, Helin Zhang, Panfeng Shang, Zizhen Hou

**Affiliations:** Department of Urology, Lanzhou University Second Hospital, Lanzhou, Gansu, China

**Keywords:** lymph node metastasis, machine learning, magnetic resonance imaging, positron emission tomography - computed tomography, prostate cancer, radiomics

## Abstract

**Background:**

This study aims to comprehensively evaluate the accuracy and efficacy of radiomics models based on imaging equipment in predicting prostate cancer (PCa) lymph node metastasis (LNM).

**Methods:**

We systematically searched PubMed, Embase, Cochrane Library, Web of Science, and Sinomed databases from their establishment until July 2024. The Quality Assessment of Diagnostic Accuracy Studies 2 (QUADAS-2) criteria and the Radiomics Quality Score (RQS) tools were utilized to assess the quality of the studies. Indicators such as the pooled area under the curve (AUC), sensitivity, specificity, positive likelihood ratio, and negative likelihood ratio were computed to evaluate the predictive effect of radiomics technology on LNM of PCa.

**Results:**

A total of 1860 patients diagnosed with LNM of PCa through histological examination were included in this meta-analysis. The radiomics model for predicting LNM in PCa showed a pooled AUC value of 0.88 (95% confidence interval (CI) [0.85 - 0.91]), with a sensitivity and specificity of 0.81 (95% CI [0.64 - 0.91]) and 0.85 (95% CI [0.75 - 0.91]), respectively. The positive likelihood ratio was 5.43 (95% CI [3.34 - 8.84]), the negative likelihood ratio was 0.22 (95% CI [0.12 - 0.43]), and the diagnostic odds ratio was 24.21 (95% CI [10.59 - 55.32]). The meta-analysis showed significant heterogeneity among the included studies. No threshold effect was detected. The subgroup analysis showed that the least absolute shrinkage and selection operator regression algorithm had the higher diagnostic sensitivity, with a pooled sensitivity of 0.96 (95% CI [0.90 - 1.00]) (*p* = 0.02), while the random forest algorithm was the opposite, with a pooled sensitivity of 0.48 (95% CI [0.16 - 0.80]) (*p* = 0.01). Radiomics features without intraclass correlation coefficient preprocessing would lead to a decrease in diagnostic specificity, 0.73 (95% CI [0.53 - 0.92]) (*p* = 0.04). The pooled specificity with an RQS score≥ 17 was 0.77 (95% CI [0.65 - 0.88]) (*p* = 0.01), and the higher the score, the lower the diagnostic specificity would be.

**Conclusions:**

The predictive model based on radiomics features has the potential to serve as an auxiliary approach for predicting preoperative LNM of PCa.

**Systematic review registration:**

https://www.crd.york.ac.uk/prospero/, identifier PROSPERO CRD42024575818.

## 1 Introduction

Prostate cancer (PCa) is the fifth most frequently diagnosed cancer worldwide, accounting for 17% of all cancer cases and ranking as the second most common cancer in men (1). Incidence rates vary significantly across regions, ranging from 6.4 to 82.8 per 100,000 individuals (1). Accurate lymph node staging is crucial for evaluating the patient’s prognosis, risk of recurrence, and potential for salvage therapy. Studies report that the recurrence rate among PCa patients with lymph node involvement at initial diagnosis ranges from 1.3% to 12%, which is closely associated with increased mortality (2). Therefore, early determination of lymph node status in PCa patients is critical (3).

Computed Tomography (CT) and Magnetic Resonance Imaging (MRI) are key modalities for detecting lymph node metastasis (LNM) in PCa, but their diagnostic accuracy remains limited (4, 5). The introduction of Positron Emission Tomography - Computed Tomography (PET-CT) has significantly enhanced accuracy by approximately 27% compared to traditional imaging equipment in detecting PCa and lymph node status. However, it also presents challenges such as reduced diagnostic sensitivity and anomalous uptake in nerve nodes (6, 7). Furthermore, the determination of lymph node status is often influenced by the spatial resolution of imaging equipment and subjective factors of the pathologist. The primary predictive models for LNM in PCa include the Memorial Sloan Kettering Cancer Center (MSKCC) model and the Briganti nomograms (2012, 2017, and 2019 editions), which aid treatment decisions, which aid treatment decisions but have limitations such as relatively low area under the curve (AUC) values and limited specificity (8–13). While pelvic lymph node dissection (Plnd) or extended pelvic lymph node dissection (Eplnd) remains the gold standard for confirming LNM, these procedures involve prolonged operative times and risks such as lymph leakage and lymphocele formation. Consequently, the indication for pelvic lymphadenectomy remains contentious.

The invasiveness of tumors is related to their heterogeneity. Radiomics technology can encode the subtle heterogeneity into quantifiable features (14, 15). By integrating these features through artificial intelligence algorithms and traditional modeling, radiomics facilitates the development of predictive models for disease status and prognosis (16). Unlike conventional histopathological biopsy, this method offers a non-invasive means of identifying the disease state and is widely utilized in medical research. Various imaging features hold potential value for evaluating the staging of PCa and lymph node status (17). Moreover, quantitative radiomic features can enhance medical decision support systems and improve clinical decision-making (18). Several studies have applied radiomics to predict LNM in PCa (19); however, the lack of standardized radiomics workflows limits model robustness and reproducibility (20).

This study aims to systematically review and comprehensively summarize existing research on the use of radiomics for evaluating LNM in PCa, focusing on diagnostic performance, sensitivity, and specificity. It seeks to provide clinicians with a potential reference tool for assessing LNM status and improving the accuracy of early diagnosis.

## 2 Methods

### 2.1 Study protocol and registration

This systematic review and meta-analysis was conducted in accordance with the Preferred Reporting Items for Systematic Reviews and Meta-Analyses (PRISMA) statement and the Quality Assessment of Diagnostic Accuracy Studies-2 (QUADAS-2) guidelines (21, 22). The protocol was registered in the International Prospective Register of Systematic Reviews (PROSPERO) database (registered number: CRD42024575818).

### 2.2 Literature search

To obtain more relevant research data, we conducted a comprehensive literature search in PubMed, Web of Science, Embase, and the Cochrane Library database, covering the time range from the establishment of each database to research published up to July 20, 2024. Additionally, the SinoMed database was searched to further ensure the inclusion of pertinent articles. During the search process, we employed a combination of Medical Subject Headings (MeSH) terms and keywords to conduct our search. The specific search terms used were as follows: (“radiomics” OR “radiomic” OR “Artificial Intelligence”[Mesh] OR “Artificial intelligence” OR “deep learning” OR “machine learning” OR “convolutional neural network” OR “automatic detection”) AND (“Magnetic Resonance Imaging”[Mesh] OR “Tomography, X-Ray Computed”[Mesh] OR “CT” OR “MRI”) AND (“Lymphatic Metastasis”[Mesh] OR “lymph node metastasis” OR “Lymph node” OR “LNM”) AND (Neoplasms, Prostatic OR Neoplasm, Prostatic OR Prostatic Neoplasm OR Prostate Neoplasms OR Neoplasms, Prostate OR Neoplasm, Prostate OR Prostate Neoplasm OR Prostate Cancer OR Cancer, Prostate OR Cancers, Prostate OR Prostate Cancers OR Cancer of Prostate OR Cancer of the Prostate OR Prostatic Cancer OR Cancer, Prostatic OR Cancers, Prostatic OR Prostatic Cancers) OR (“Prostatic Neoplasms”[Mesh]). The specific search strategies implemented in each database are detailed in **Supplementary S1**.

### 2.3 Literature screening

A rigorous screening process was implemented to remove duplicate records from the initial dataset. Subsequently, titles and abstracts were thoroughly reviewed. To address selective reporting bias, two authors (YH.Z. and YL.D.) independently assessed the abstracts and titles to determine which studies met the inclusion criteria for full-text review. Discrepancies in study selection were resolved through consultation with a third reviewer(the corresponding author, PF.S.). By adhering to the PICO standard and formulating a specific literature search strategy, we ensured an exhaustive and impartial search as follows:

P (Population): Patients who underwent radical prostatectomy combined with pelvic lymph node dissection and were affirmed to have PCa through histopathological examination. I (Intervention): Prior to the diagnosis of PCa, CT and MRI imaging examinations were undergone. C (Comparator): Histopathologic results were used as the reference standard to compare the performance of radiomics models. O (Outcomes): The performance of Radiomics models was assessed through key metrics, including AUC, sensitivity, specificity, positive and negative likelihood ratios, and diagnostic odds ratios.

The exclusion criteria are as follows (1): Irrelevant titles and abstracts; (2) Unqualified publication types, such as case reports, review articles, editorials, letters, errata, conference abstracts, and animal experiments. All studies that fail to comply with these criteria were excluded to ensure the reliability and quality of the meta-analysis data.

### 2.4 Data extraction

The data extraction for the study was conducted independently by two authors (YH.Z. and YL.D.), who utilized WPS Office software (version 6.10.1) to record the data on an electronic spreadsheet. Any discrepancies were resolved through consultation with the corresponding author (PF.S.). The extracted data encompassed: (1) General study information (first author’s name, publication year, country); (2) Parameters related to radiomics techniques (imaging equipment, tumor lesion segmentation method, region of interest (ROI) dimensions, imaging feature extraction software, imaging feature types); (3) Details about the development and validation of the prediction model (clinical characteristics including the number of patients, the number of lymph nodes, positive rate of lymph nodes, lymph node dissection procedure, study design, number of centers; Intraclass Correlation Coefficient (ICC) or not; standardization or not; classifier model; and model validation method); (4) Performance evaluation indicators for the prediction model such as AUC value, sensitivity, specificity along with their respective 95% confidence intervals (95% CI) as well as true positives (TP), false positives (FP), true negatives (TN), and false negatives (FN). The AUC value is derived from the highest validation set or test set of the predictive model developed based on radiomics features. For single-center studies, the AUC value stems from the validation set or the test set. If multi-center data exist in the study, the result with the highest AUC value from the external validation set will be incorporated.

### 2.5 Quality assessment

The Radiomics Quality Score (RQS) checklist and QUADAS-2 were employed to evaluate the included studies (22, 23). Two authors (YH.Z. and YL.D) independently conducted the assessments, with any discrepancies resolved through consultation with the corresponding author (PF.S.). The RQS checklist, proposed by Lambin et al. in 2017, is a specialized tool for assessing the quality of radiomics research. It evaluates 16 components across six key domains to measure the methodological rigor of the radiomics workflow. Complementing the radiomics focus, the QUADAS-2 tool addresses issues related to applicability and bias risk in diagnostic accuracy studies. The details of each study can be found in **Supplementary S2**, **Supplementary S3**.

### 2.6 Statistical analysis

All statistical analysis and graphical representations were performed using STATA (Version 18.0), R Studio (version 4.3.1), and Origin pro (Version 2022) incorporating the R packages “metamisc” and “metaphor”. Summary receiver operating characteristic (SROC) curves were constructed from 2 × 2 contingency table data to evaluate diagnostic test performance. The area under the curve (AUC) was used as a metric to assess the predictive models’ accuracy. Diagnostic metrics including sensitivity, specificity, positive likelihood ratio, negative likelihood ratio, diagnostic odds ratio, and diagnostic score were calculated with their corresponding 95% confidence intervals. Missing data were estimated using either the confusion matrix calculator or R Studio-based methods. Detailed formulas and procedures can be found in **Supplementary S4**.

The Q-test and I^2^ statistic were combined to assess heterogeneity among study results, with heterogeneity classified as very low (0–25%), low (25–50%), moderate (50–75%), and high (>75%). Based on the degree of heterogeneity, either a fixed-effect or random-effects meta-analysis model was employed.

In subgroup analysis, multiple covariates were evaluated to determine the source of heterogeneity, including whether clinical characteristics, calibration method of model, study design, imaging equipment, tumor lesion segmentation method, ROI dimension, imaging feature extraction software, lymph node dissection procedure, ICC or not, standardization or not, classifier model, and model validation method, and classification based on the median RQS score as RQS ≥ median or not.

Continuous numerical variables were also examined in the meta-analysis as potential sources of heterogeneity, including the number of patients, size of validation cohorts, number of lymph node-positive cases, and lymph node positivity rate (a total of four items).

A stepwise sensitivity analysis was conducted by sequentially omitting one study at a time to evaluate the influence of individual studies on the overall estimate.

The Deek’s funnel plot was utilized to examine potential publication bias, while the Egger’s test quantitatively evaluated the risk of such bias. Additionally, we applied the Fagan plot to assess clinical utility by providing pre-test probabilities for LNM when calculating post-test probabilities. Statistical significance was defined as P < 0.05.

## 3 Results

### 3.1 Study screening and selection

**Figure 1:**
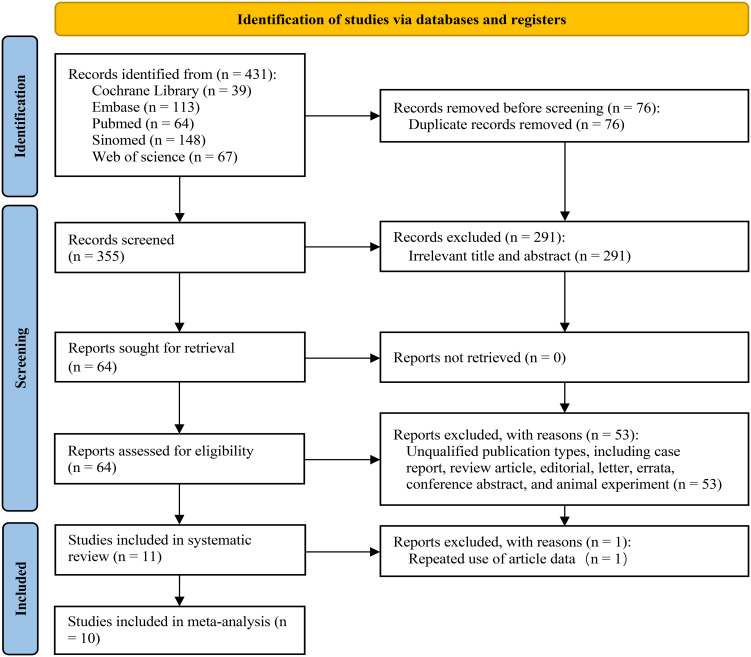
The PRISMA flowchart for the study screening process.

Through our systematic search strategy, we identified 431 studies from 5 databases. Following the removal of duplicate studies, 355 records remained for subsequent screening. Upon the review of the titles and abstracts, 291 documents were excluded due to non-compliance with the PICO criteria. Subsequently, a detailed screening and evaluation were conducted on 64 articles. Among these, 53 articles were eliminated as they failed to comply with the requirements of the literature type. Ultimately, 11 articles that conformed to the PICO criteria and whose full texts were accessible were incorporated into the systematic review; nonetheless, 1 article was precluded from further meta-analysis on account of data reuse (24), thus a total of 10 articles were included in the meta-analysis (19, 25–33). **Figure 1** presents a comprehensive depiction of the entire literature inclusion process.

### 3.2 Study characteristics and data

These 11 articles were published between 2019 and 2023, involving a total of 1,931 patients (**Table 1**). In the included studies, there were 5 from Europe, 5 from Asia, and 1 from North America. It is worth noting that all the Asian literature originated from China. All the suspected metastatic lymph nodes were resected by Plnd and/or Eplnd method before the operation and confirmed histopathologically as LNM of PCa. Most of the studies were retrospective in design, with only 1 being prospective. Only 2 studies had multi-center data sources, while 9 were from a single center. In the radiomics workflow, 7 studies used imaging equipment MRI to obtain the original images, while the rest used PET-CT. In tumor lesion segmentation, manual segmentation is commonly employed to define the tumor dimension in three-dimensional space. 7 studies utilized the open-source software PyRadiomics for feature extraction, with all studies extracting representative texture features. Furthermore, Bourbonne et al. and Lai et al. conducted ICC evaluations of radiomics features to ensure imaging feature accuracy (19, 28). 6 studies standardized the extracted imaging feature values during data processing. For model building and validation, machine learning algorithms were used in 6 studies, traditional linear algorithms in 4 studies, and deep learning (DL) algorithms in only 1. To enhance model robustness, 7 studies employed cross-validation while 2 used bootstrapping methods; however, 2 did not specify the validation method. Internal validation was predominantly utilized, although Hou et al., Luining et al., and Peeken et al. incorporated external validation methods to bolster model reliability (26, 27, 31).

**Table 1:** The table of the characteristics and data included in the study.

Author	Year	TP	FP	FN	TN	AUC	Sensitivity	Specificity	LCI	UCI	Number of patients	Number of validation set	Number of lymph nodes positive	Positive rate of lymph nodes	RQS scores ≥17
Bourbonne	2021	16	5	3	88	0.89	0.65	0.91	/	/	280	112	19	0.17	Yes
Cysouw	2021	8	14	2	38	0.86	0.82	0.85	/	/	72	14	10	0.16	Yes
Hou	2021	3	6	3	38	0.78	0.86	1	0.65	0.88	401	50	6	0.12	No
Lai	2021	6	1	1	5	0.89	1	0.46	0.8	0.97	42	13	7	0.54	No
Liu	2022	14	42	3	69	0.73	0.79	0.9	0.65	0.81	474	128	17	0.13	Yes
Liu-2	2022	38	27	5	138	0.9	0.88	0.84	0.85	0.94	84	208	43	0.21	Yes
Liu-3	2022	12	0	2	31	0.96	0.857	1	0.86	0.99	71	45	14	0.31	Yes
Luining	2023	2	5	9	35	0.57	0.5	0.86	0.4	0.75	123	51	11	0.22	No
Peeken	2021	49	7	0	6	0.95	0.84	0.74	0.88	0.99	80	62	49	0.79	Yes
Zamboglou	2019	12	2	7	19	0.85	0.81	0.62	0.74	0.96	60	40	19	0.48	No
Zheng	2022	24	5	7	48	0.92	0.18	0.88	0.85	0.98	244	84	31	0.37	No
Nation location	Clinical characteristics	Center	Study design	Calibration method of model	Imaging equipment	Lymph node dissection procedure	Model validation method	Classifier model	Imaging feature extraction software	Segmentation method	ROI dimension	ICC	Standardization		
France	Yes	Single	Retrospective	Fold- bootstraping	MRI	Eplnd	Internal	DL	PyRadiomics	Semi-automatical	3D	Yes	No		
Netherlands	Yes	Single	Prospective	Cross-validation	PET-CT	Eplnd	Internal	RF	RaCat	Manual	3D	No	Yes		
China	Yes	Multiple	Retrospective	Cross-validation	MRI	Eplnd	External	RF	PyRadiomics	Manual	2D	Else	Yes		
China	No	Single	Retrospective	Unclear	MRI	Eplnd	Internal	LR	Others	Manual	2D	Yes	No		
China	No	Single	Retrospective	Cross-validation	MRI	Eplnd	Internal	SVM	PyRadiomics	Automatic	3D	Yes	Yes		
China	No	Single	Retrospective	Cross-validation	MRI	Combined	Internal	LASSO	PyRadiomics	Automatic	3D	Yes	Yes		
China	No	Single	Retrospective	Cross-validation	MRI	Eplnd	Internal	RF	PyRadiomics	Manual	3D	No	Yes		
Netherlands	No	Multiple	Retrospective	Cross-validation	PET-CT	Eplnd	External	RF	RaCat	Semi-automatical	3D	Yes	Yes		
Germany	No	Multiple	Retrospective	Fold- bootstraping	PET-CT	Eplnd	External	LASSO	PyRadiomics	Manual	3D	No	No		
Germany	No	Single	Retrospective	Unclear	PET-CT	Plnd	Internal	LR	Others	Manual	3D	No	No		
California	Yes	Single	Retrospective	Cross-validation	MRI	Combined	Internal	SVM	PyRadiomics	Manual	3D	Yes	Yes		

2D, two-dimensional; 3D, three-dimensional; AUC, Area Under Curve; CI, Confidence Interval; CT, Computed Tomography; DL, Deep Learning; Eplnd, Extended pelvic lymph node dissection; FN, false negatives; FP, false positives; ICC, Intraclass Correlation Coefficient; LASSO, the least absolute shrinkage and selection operator regression; LCI, Lower Confidence Interval; LR, Logistic Regression; MRI, magnetic resonance imaging; PET-CT, Positron Emission Tomography-Computed Tomography; Plnd, pelvic lymph node dissection; ROI, reign of interest; RF, Random Forest; SVM, Support Vector Machine; TN, true negatives; TP, true positives; UCI, Upper Confidence Interval.

### 3.3 Data quality assessment

**Figure 2:**
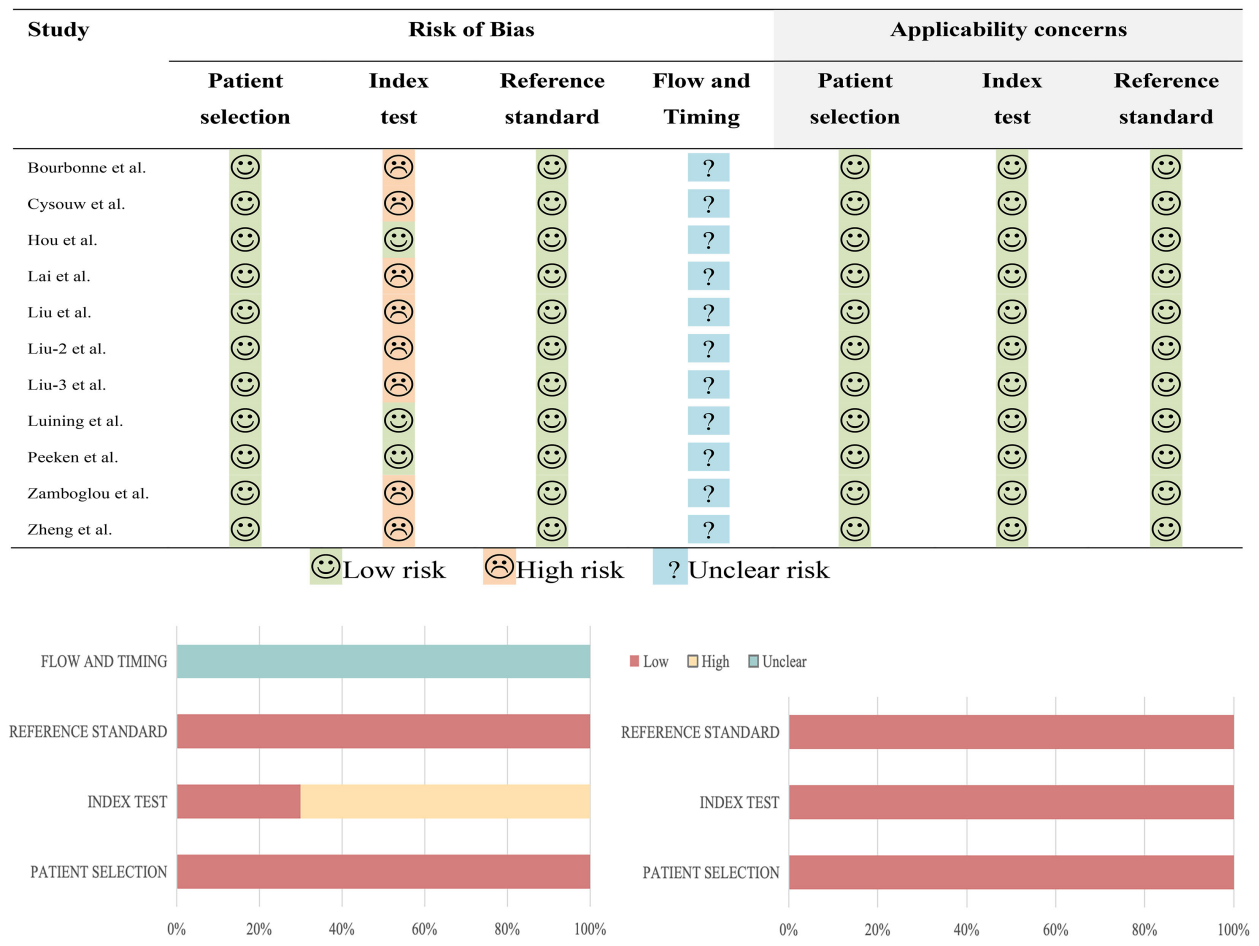
The summary of the quality assessment of the included study following QUADAS-2.

Upon utilizing the QUADAS-2 tool, it was discerned that none of the studies exhibited a low risk of bias and practical relevance. 8 studies exhibited a high risk of bias in the domain of test selection, while 11 studies showed a low risk of bias in both patient selection and reference standard domains. The risk of bias in the flow and time domains remained uncertain in 11 studies, primarily due to inadequate reporting of the interval between the index test and reference standard test. In terms of applicability concerns, all studies were deemed to pose low risk. The specific details are depicted in **Figure 2**.

**Figure 3:**
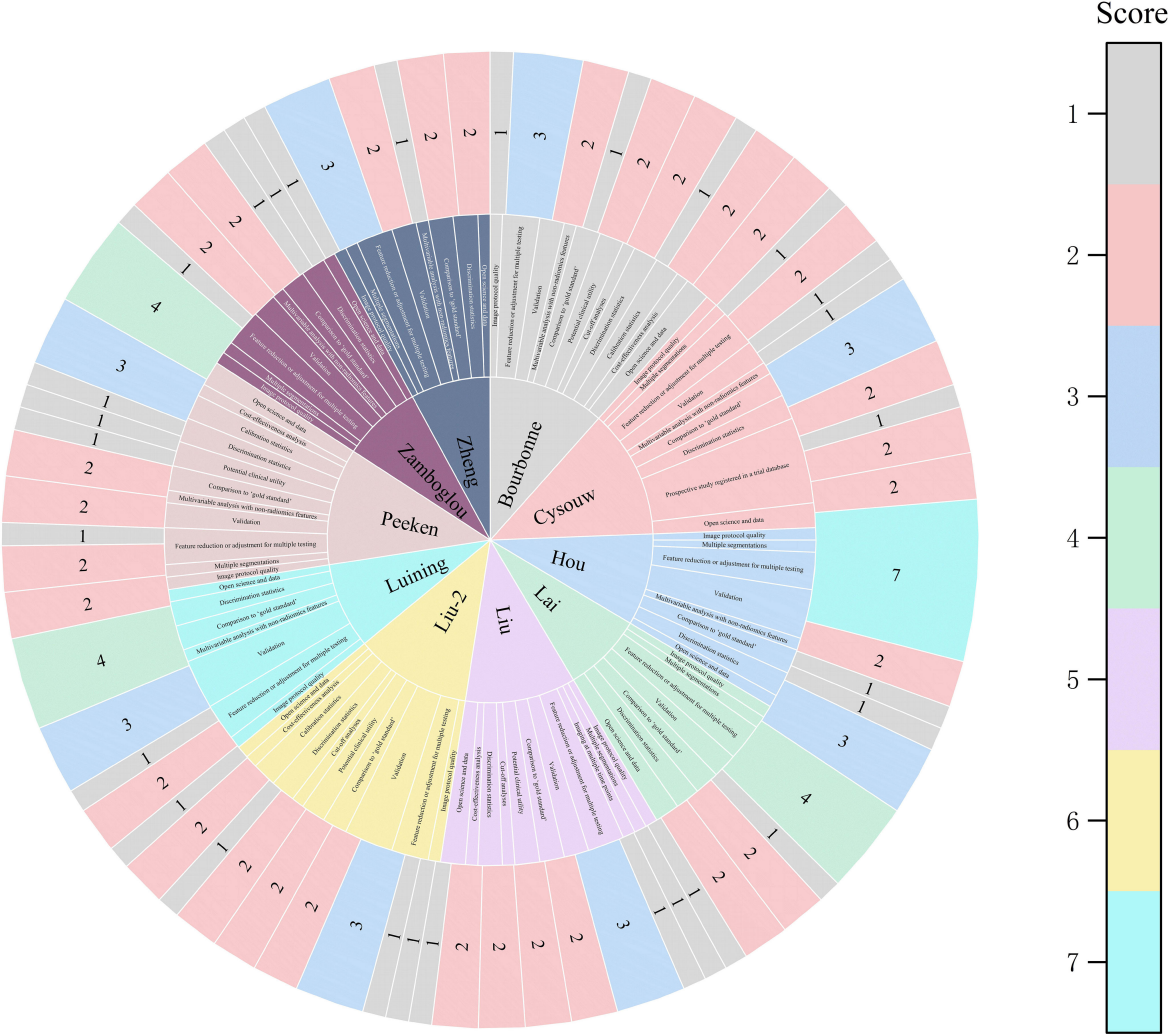
The figure of the RQS scores of the studies included in the meta-analysis. The diverse color scales on the right side of the figure denote distinct scores. The scores ascend from top to bottom. The project score of 0 is not presented in the figure.

Among the 11 studies included in the systematic review, the range of RQS scores was 13 to 21 (**Table 2**); and among the 10 studies included in the meta-analysis, the range of RQS scores was also 13 to 21, with an average ± standard deviation of 16.4 ± 2.94 and a median of 16.5 (**Figure 3**).

**Table 2:** The table of RQS scores for each study.

Author/Year	Domain (Score)						Total scores
	Domain 1	Domain 2	Domain 3	Domain 4	Domain 5	Domain 6	
Bourbonne et al. (19) 2021	1	5	5	5	1	2	19
Cysouw et al. (25) 2021	2	5	3	2	7	2	21
Hou et al. (27) 2021	2	7	3	2	0	1	15
Lai et al. (28) 2021	2	5	2	2	0	2	13
Liu et al. (30) 2022	3	5	4	3	1	2	18
Liu-2 et al. (29) 2022	1	7	4	5	1	1	19
Liu-3 et al. (24) 2022	3	5	5	4	1	1	19
Luining et al. (26) 2023	1	7	3	2	0	1	14
Peeken et al. (31) 2021	2	5	5	4	1	2	19
Zamboglou et al. (32) 2019	2	5	3	2	0	1	13
Zheng et al. (33) 2022	2	5	3	2	0	1	13

### 3.4 Data analysis

Through analyzing and combining the results of diagnostic indicators, it was shown that the prediction model developed based on radiomics technology had good diagnostic performance in predicting LNM in PCa patients preoperatively. AUC was 0.88 (95% CI [0.85 - 0.91]), sensitivity was 0.81 (95% CI [0.62 - 0.91]), specificity was 0.83 (95% CI [0.73 - 0.90]), positive likelihood ratio (PLR) was 4.69 (95% CI [3.11 - 7.10]), negative likelihood ratio (NLR) was 0.23(95% CI [0.11-0.48]), diagnostic odds ratio (DOR) was 20(95% CI [9 - 45]), diagnostic Score was 3 (95% CI [2.19 - 3.81]).

**Figure 4:**
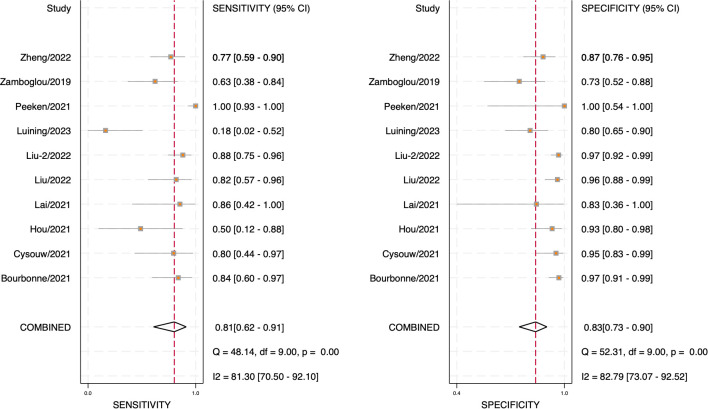
Forest plot of sensitivity and specificity.

**Figure 5:**
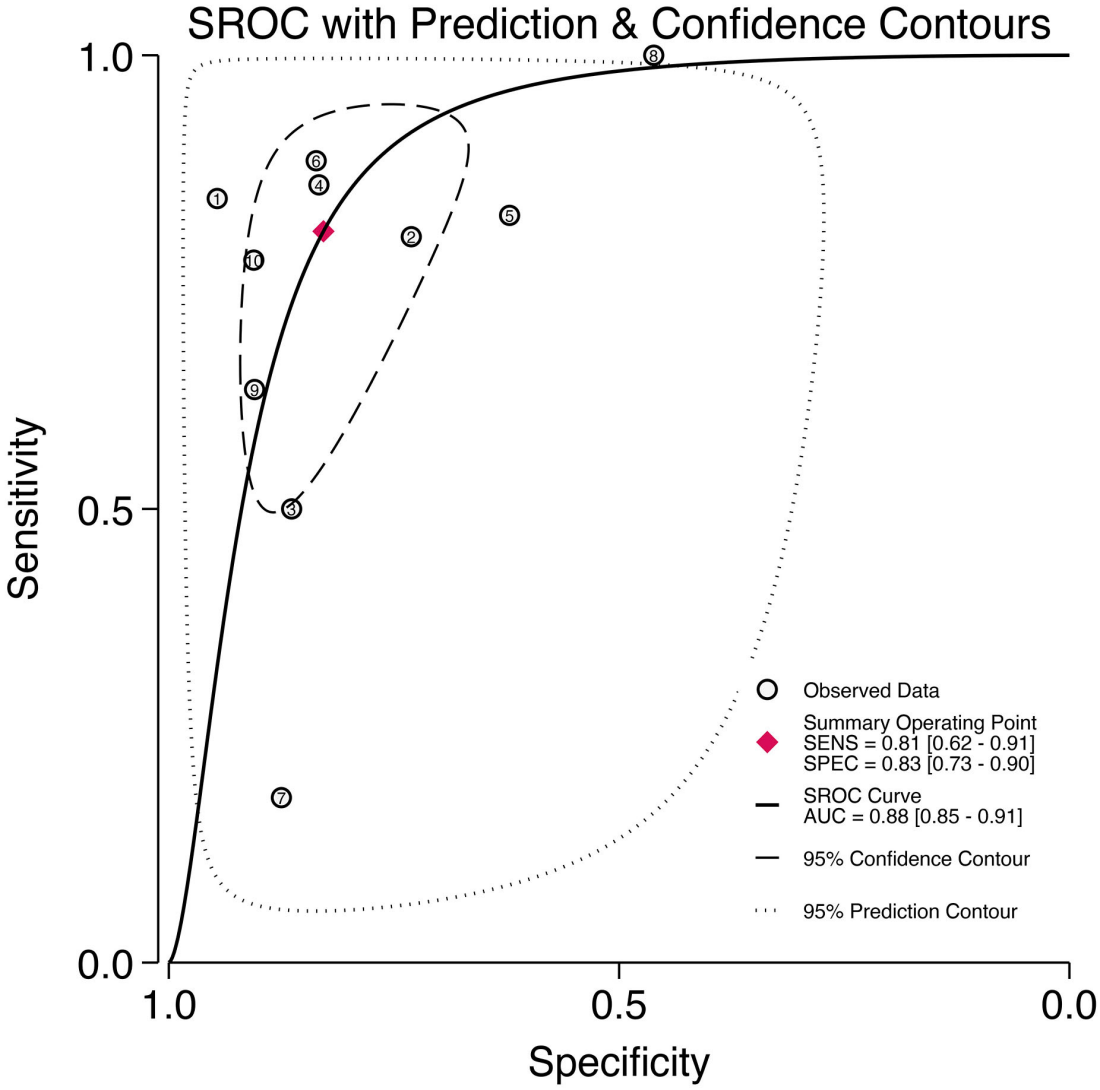
The SROC curve for the prediction of lymph node metastasis of prostate cancer based on radiomics technology.

The forest plot illustrating the combined sensitivity and specificity is depicted in **Figure 4**, while the SROC curve is presented in **Figure 5**. Detailed information on diagnostic likelihood ratios, diagnostic scores, and diagnostic odds ratios can be found in **Supplementary S5**, **Supplementary S6**.

### 3.5 Heterogeneity test

The results of Cochran’s Q and Higgins I^2^ tests indicate a high level of heterogeneity in pooled sensitivity and specificity, with Q values of 52.31 (*p* < 0.01) and I^2^ values of 82.79 for sensitivity, as well as Q values of 48.14 (*p* < 0.01) and I2 values of 81.30% for specificity. The Spearman correlation coefficient of 0.45 (*p* > 0.05) suggests the absence of a threshold effect.

### 3.6 Subgroup analysis

The subgroup analysis revealed that the Least Absolute Shrinkage and Selection Operator regression (LASSO) algorithm significantly enhanced diagnostic sensitivity, yielding a combined sensitivity of 0.96 [0.90 - 1.00] (*p*=0.02), whereas the random forest (RF) algorithm had an adverse effect, resulting in a combined sensitivity of 0.48 [0.16 - 0.80] (p=0.01). Imaging features not selected by the ICC led to a reduction in diagnostic specificity, resulting in a combined specificity of 0.73 (0.53 - 0.92) (*p*=0.04). The combined specificity of RQS score ≥ 17 was 0.77 (0.65 - 0.88) (p=0.01), and higher RQS scores were associated with lower diagnostic specificity. In addition, the combination of radiomics technology with clinical characteristics, calibration method of model, study design, imaging feature extraction software, imaging equipment, lymph node dissection procedure, model validation method, segmentation method, ROI dimension, and Standardization did not exhibit statistically significant effects on sensitivity and specificity. Prospective and retrospective designs demonstrated similar discriminatory ability in prediction models. Moreover, differences between PyRadiomics software and other feature extraction software in prediction models were found to be nonsignificant.

Incorporating clinical characteristics, Fold-bootstraping, LASSO classifier; uncalculated ICC values; combined Plnd with Eplnd; internal validation method; 3D tumor lesions; RQS score ≥ 17; manual segmentation; data Standardization; and Fold-bootstrap method all contributed to improved pooled AUC value of prediction models. However, there was no statistically significant difference in the above results. For further details please refer to **Table 3** and **Supplementary S7**, **Supplementary S8**.

**Table 3:** The table of subgroup analysis results in the included study.

Characteristic	Category	Number of studies	Sensitivity (95% CI)	*p*1	Specificity (95% CI)	*p*2	AUC (95% CI)	*p*3
Clinical characteristics	Yes	4	0.78 [0.53 - 1.00]	0.73	0.88 [0.80 - 0.96]	0.75	0.88 [0.79 - 0.93]	0.00
	No	6	0.83 [0.65 - 1.00]	/	0.88 [0.80 - 0.96]	/	0.81 [0.62 - 0.92]	0.00
Calibration method of model	Fold-bootstraping	2	0.97 [0.91 - 1.00]	0.01^*^	0.81 [0.62 - 1.00]	0.47	0.89 [0.78-0.95]	0.00
	Cross validation	6	0.72 [0.49 - 0.96]	0.19	0.82 [0.72 - 0.93]	0.20	0.83 [0.70-0.91]	0.00
	unclear	2	0.76 [0.38 - 1.00]	0.94	0.89 [0.72 - 1.00]	0.68	0.87 [0.37-0.99]	0.13
Classifier model	DL	1	0.70 [0.48 - 0.93]	0.09	0.85 [0.76 - 0.94]	0.6	0.89 [0.78 - 0.95]	0.00
	LASSO	2	0.96 [0.90 - 1.00]	0.02^*^	0.70 [0.46 - 0.94]	0.07	0.91 [0.63 - 0.99]	0.01
	LR	2	0.76 [0.38 - 1.00]	0.94	0.89 [0.72 - 1.00]	0.68	0.87 [0.37 - 0.99]	0.13
	RF	3	0.48 [0.16 - 0.80]	0.01^*^	0.84 [0.70 - 0.99]	0.48	0.83 [0.66 - 0.93]	0.00
	SVM	2	0.82 [0.53 - 1.00]	0.75	0.78 [0.58 - 0.98]	0.23	0.77 [0.45 - 0.94]	0.10
Study design	Prospective	1	0.82 [0.36 - 1.00]	0.52	0.73 [0.41 - 1.00]	0.41	0.86 [0.68 - 0.95]	0.00
	Retrospective	9	0.80 [0.65 - 0.96]	/	0.84 [0.76 - 0.92]	/	0.86 [0.77 - 0.92]	0.00
ICC	Yes	6	0.78 [0.57 - 0.98]	0.61	0.86 [0.77 - 0.94]	0.75	0.86 [0.76 - 0.92]	0.00
	No	3	0.90 [0.75 - 1.00]	0.25	0.73 [0.53 - 0.92]	0.04^*^	0.87 [0.70 - 0.95]	0.00
	Others	1	0.50 [-0.24 - 1.00]	0.33	0.87 [0.67 - 1.00]	0.76	0.78 [0.20 - 0.98]	0.35
Imaging feature extraction software	PyRadiomics	6	0.88 [0.77 - 0.98]	0.2	0.82 [0.71 - 0.92]	0.26	0.87 [0.78 - 0.93]	0.00
	RaCat	2	0.48 [0.06 - 0.91]	0.06	0.82 [0.64 - 1.00]	0.52	0.84 [0.65 - 0.93]	0.00
	Others	2	0.76 [0.38 - 1.00]	0.94	0.89 [0.72 - 1.00]	0.68	0.87 [0.37 - 0.99]	0.13
Imaging equipment	PET-CT	4	0.77 [0.53 - 1.00]	0.68	0.77 [0.62 - 0.93]	0.08	0.84 [0.68 - 0.93]	0.00
	MRI	6	0.82 [0.66 - 0.99]	/	0.85 [0.76 - 0.94]	/	0.87 [0.77 - 0.93]	0.00
Lymph node dissection procedure	Plnd	1	0.64 [0.02 - 1.00]	0.63	0.91 [0.74 - 1.00]	0.31	0.85 [0.16 - 0.99]	0.32
	Eplnd	7	0.81 [0.63 - 0.99]	0.92	0.79 [0.69 - 0.90]	0.04	0.85 [0.77 - 0.91]	0.00
	Combined	2	0.84 [0.60 - 1.00]	0.63	0.87 [0.75 - 1.00]	0.86	0.91 [0.63 - 0.98]	0.01
Model validation method	External	3	0.72 [0.39 - 1.00]	0.54	0.78 [0.59 - 0.97]	0.19	0.76 [0.35 - 0.95]	0.21
	Internal	7	0.83 [0.68 - 0.98]	/	0.84 [0.75 - 0.94]	/	0.87 [0.79 - 0.92]	0.00
ROI dimension	3D	8	0.82 [0.67 - 0.97]	0.43	0.82 [0.73 - 0.91]	0.56	0.87 [0.78 - 0.92]	0.00
	2D	2	0.72 [0.27 - 1.00]	/	0.85 [0.66 - 1.00]	/	0.83 [0.37 - 0.98]	0.15
RQS score	≥17	5	0.91 [0.83 - 0.99]	0.1	0.77 [0.65 - 0.88]	0.01^*^	0.87 [0.78 - 0.91]	0.00
	<17	5	0.61 [0.40 - 0.83]	/	0.89 [0.81 - 0.97]	/	0.80 [0.50 - 0.94]	0.05
Segmentation method	Manual	6	0.84 [0.68 - 1.00]	0.58	0.81 [0.69 - 0.93]	0.14	0.86 [0.73 - 0.94]	0.00
	Semi-automatical	2	0.55 [0.10 - 1.00]	0.14	0.93 [0.85 - 1.00]	0.68	0.85 [0.56 - 0.96]	0.02
	Automatic	2	0.88 [0.66 - 1.00]	0.42	0.74 [0.54 - 0.95]	0.09	0.81 [0.56 - 0.94]	0.02
Standardization	Yes	6	0.72 [0.49 - 0.96]	0.19	0.82 [0.72 - 0.93]	0.2	0.83 [0.70 - 0.91]	0.00
	No	4	0.91 [0.78 - 1.00]	/	0.86 [0.73 - 0.98]	/	0.89 [0.79 - 0.95]	0.00

2D, two-dimensional; 3D, three-dimensional; AUC, Area Under the Curve; CI, Confidence Interval; CT, Computed Tomography; DL, Deep Learning; Eplnd, Extended pelvic lymph node dissection; ICC, Intraclass Correlation Coefficient; LASSO, Least Absolute Shrinkage and Selection Operator; LR, Logistic Regression; MRI, magnetic resonance imaging; PET-CT, Positron Emission Tomography-Computed Tomography; Plnd, pelvic lymph node dissection; ROI, region of interest; RF, Random Forest; SVM, Support Vector Machine; *p*1/*p*2, Cochran’s Q-test was used to compare sensitivity/specificity between subgroups; *p*3: Wald test from meta-regression was used to compare AUC between subgroups.*, statistically significant difference.

### 3.7 Sensitivity analysis

**Figure 6:**
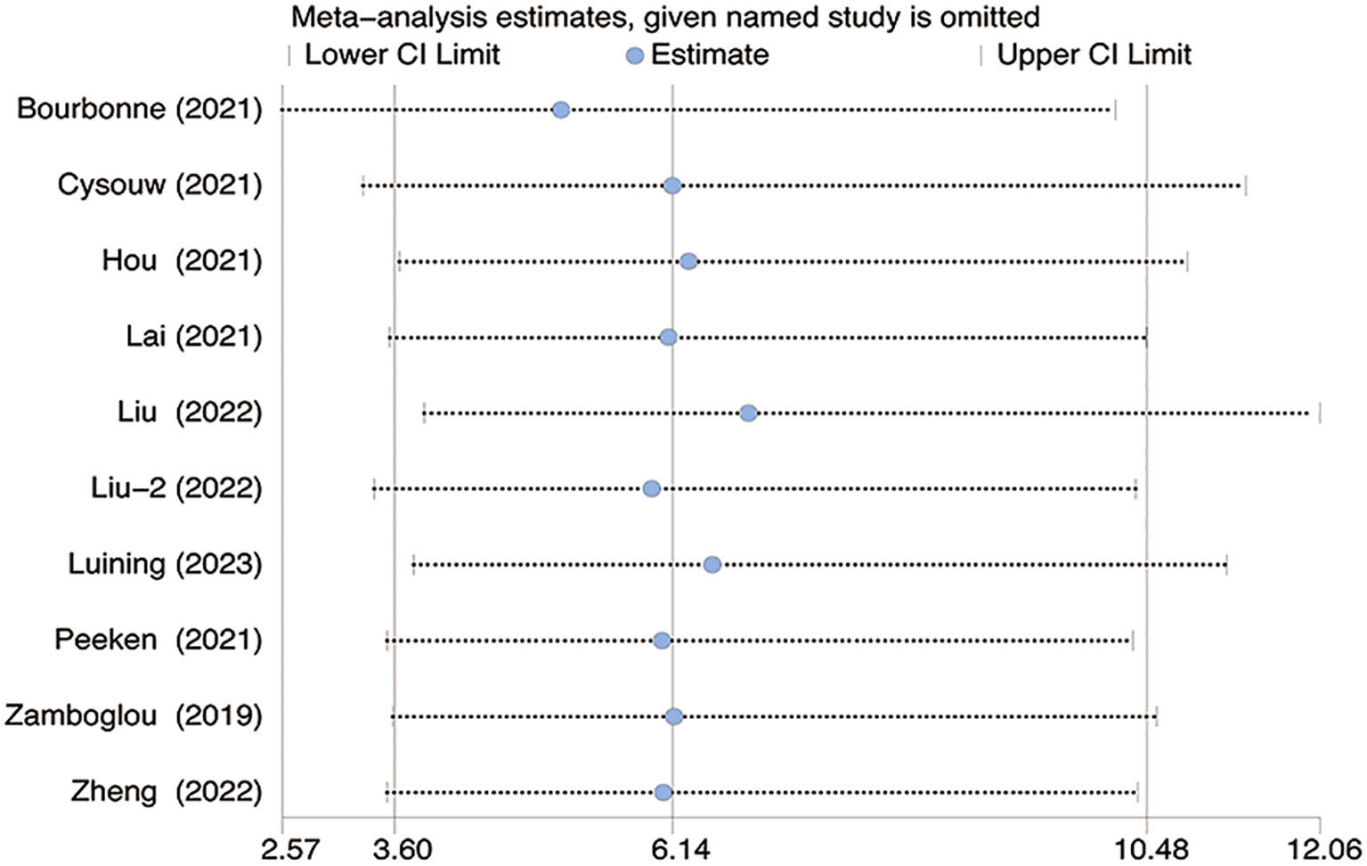
The figure for sensitivity analysis was calculated using the stepwise rejection method. CI, Confidence Interval.

The sensitivity analysis revealed no significant changes upon systematically removing one study at a time as shown in **Figure 6**.

### 3.8 Meta-regression

**Figure 7:**
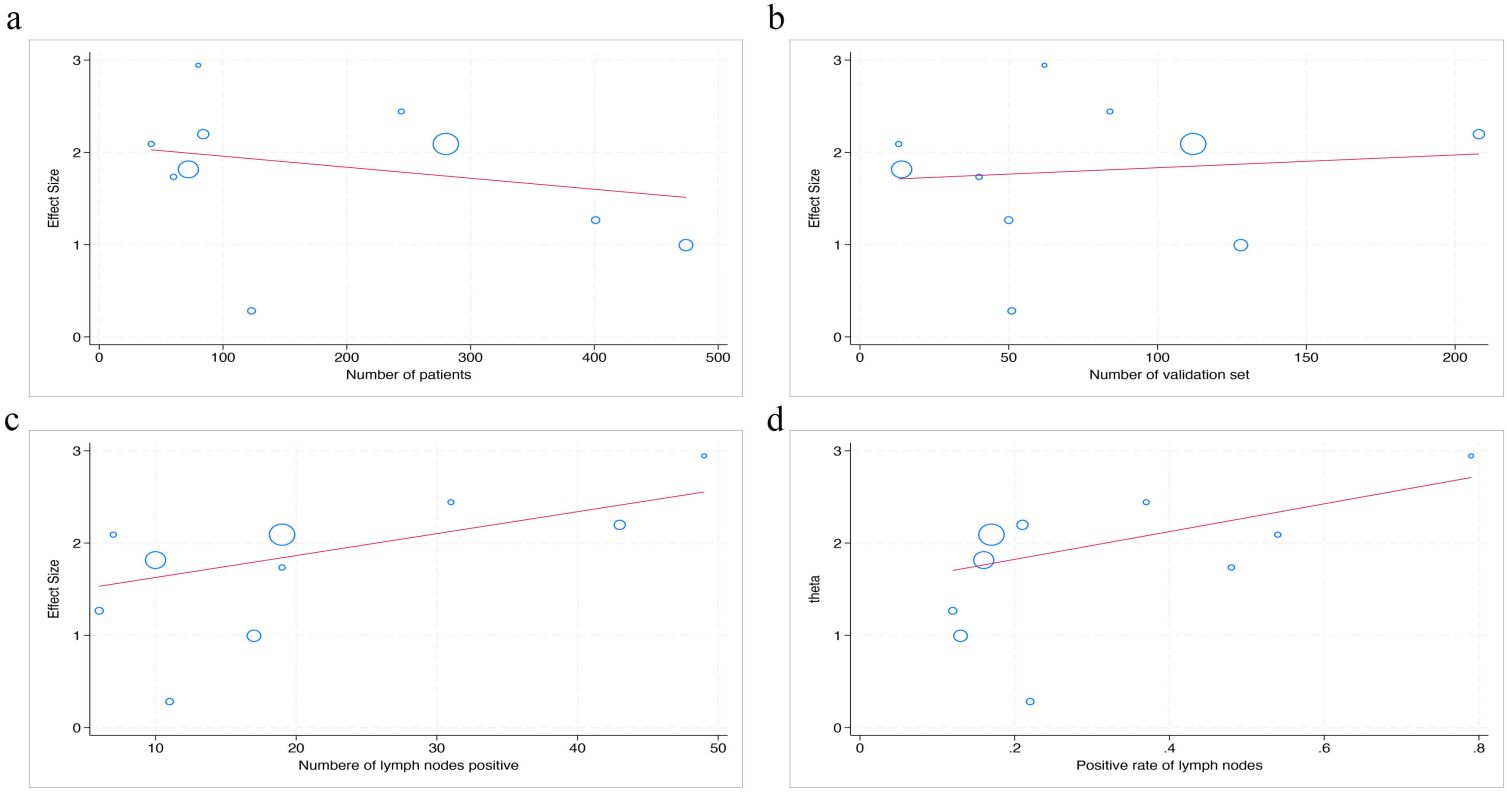
The figure of meta-regression between AUC value and numerical variables. The number of patients **(a)**, the number of validation set **(b)**, the number of lymph nodes positive **(c)**, and the positive rate of lymph nodes **(d)** (*p* = 0.56, *p* = 0.78, *p* = 0.43, *p* = 0.57, respectively).

The statistical results show that there is no significant difference in AUC value between the number of patients, the number of validation set, the number of lymph nodes positive, and the positive rate of lymph nodes (p=0.56; p=0.78; p=0.43; p=0.57) (**Figure 7**).

### 3.9 Publication bias

**Figure 8:**
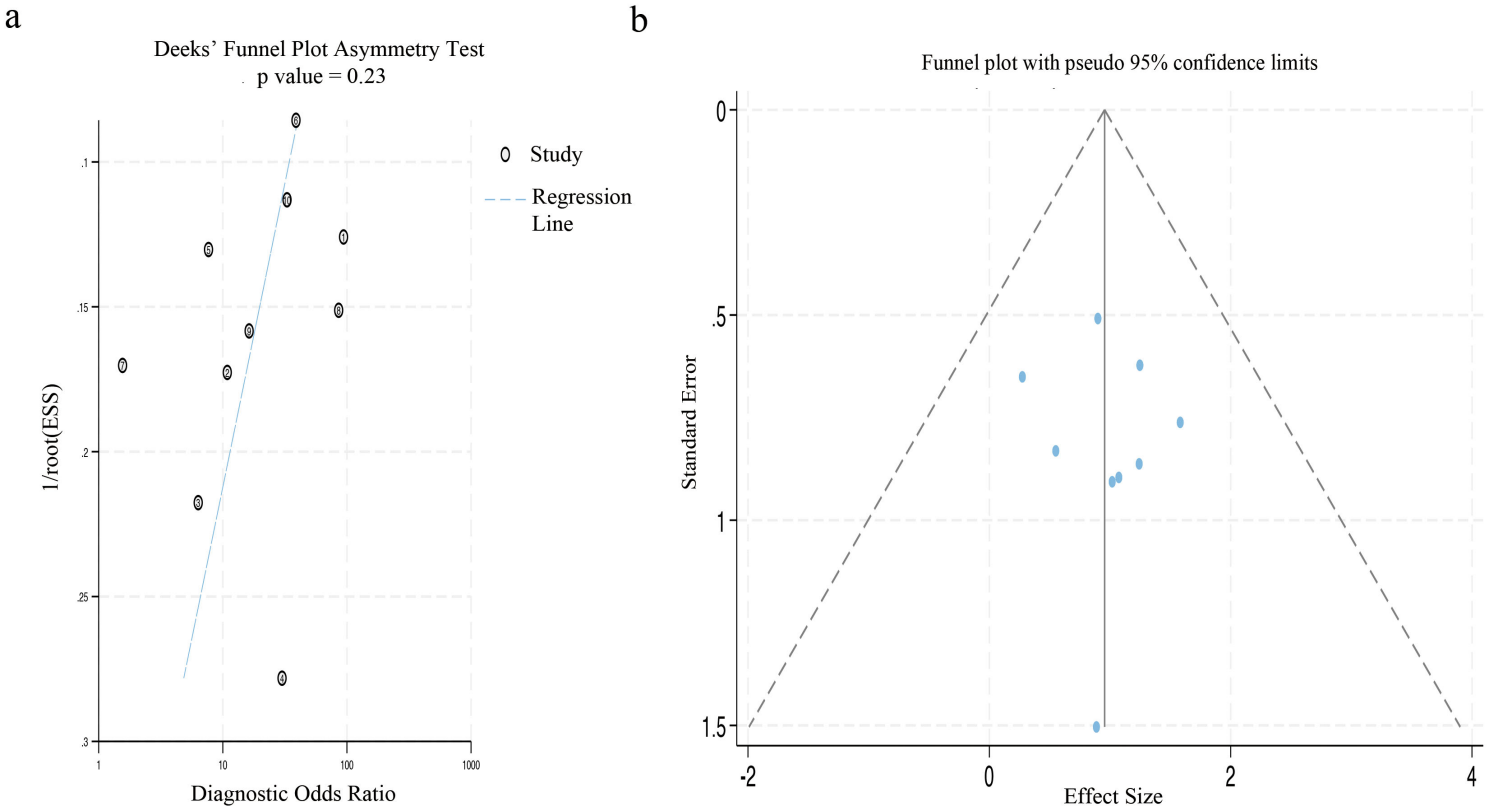
The figure of publication bias using Deek’s test and Egger’s test. **(a)** The funnel plot for publication bias was assessed by applying Deek’s asymmetry test. **(b)** This funnel plot presented all the studies encompassed in the meta-analysis, and each point represented an independent study.

The Deek’s funnel plot asymmetry test did not reveal a statistically significant bias (p=0.23). Similarly, the results of Egger’s test indicated no substantial publication bias in the included studies (p=0.613), as illustrated in **Figure 8**.

### 3.10 Clinical utility

**Figure 9:**
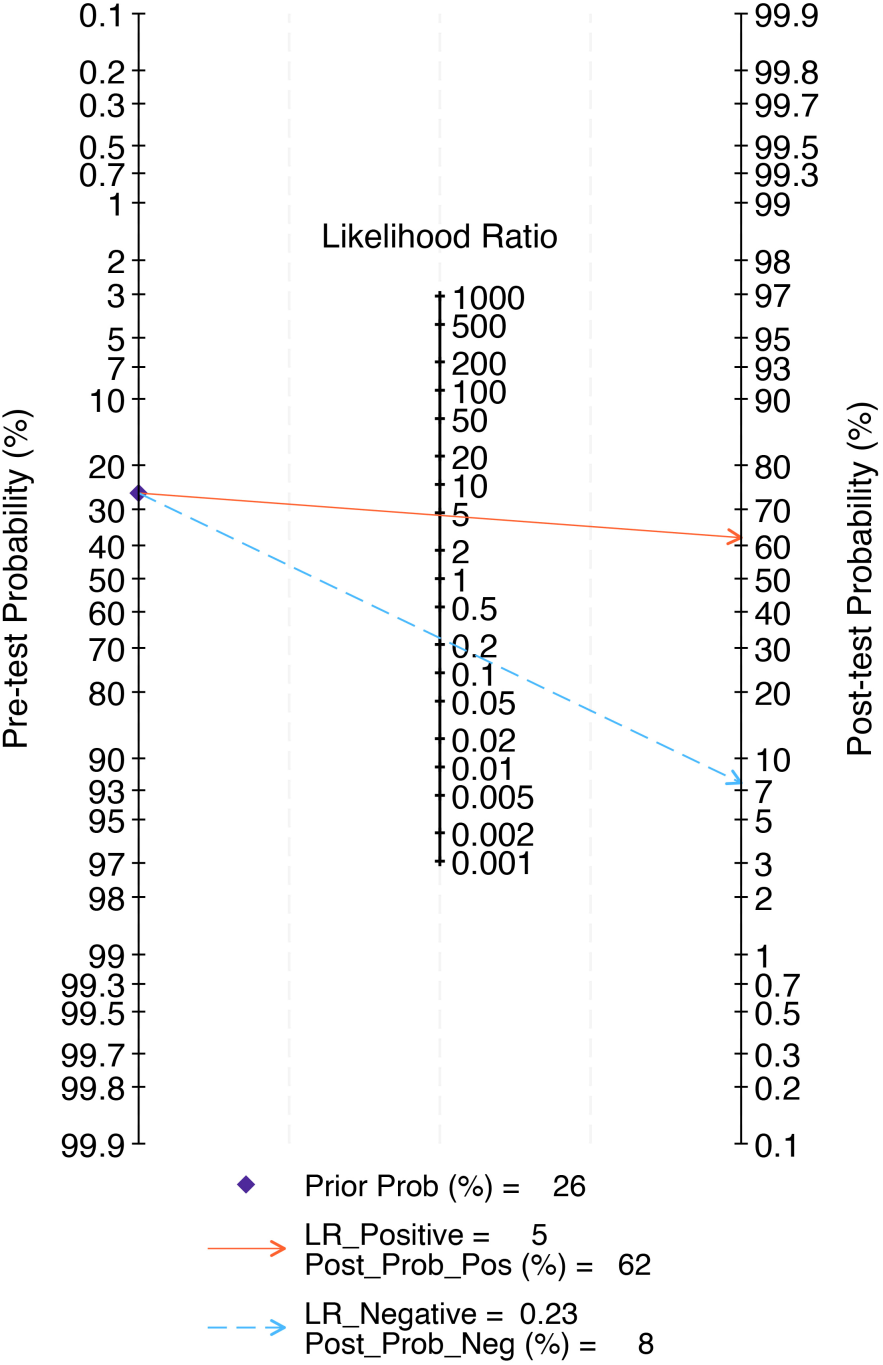
The Fagan nomogram of the radiomics model in the detection of lymph node metastasis of prostate cancer. The Fagan nomogram demonstrated the performance of the radiomics model in the detection of lymph node metastasis in prostate cancer. The pre-test probability of having lymph node metastasis was 26%, yielding a post-test probability of 62% with a positive test and 8% with a negative test.

The combined findings indicate that the pre-test probability is 26%. Furthermore, the positive predictive value of the LNM test-based radiomics model prediction is 62%, while the negative predictive value is 8%, as illustrated in **Figure 9**.

## 4 Discussion

This study presents a systematic review and meta-analysis of ten studies that developed radiomics-based predictive models for LNM in prostate in PCa. To our knowledge, this is the first comprehensive evaluation of radiomics technology in predicting LNM in PCa. The pooled results indicate that the models demonstrated a good performance in predicting LNM (0.88 [95% CI: 0.85 - 0.91]) with high sensitivity (0.81 [95% CI: 0.62 - 0.91]) and specificity (0.83 [95% CI: 0.73 - 0.90]). Using radiomics - based predictive models to predict LNM of PCa before surgery may provide certain reference value for clinical decision-making.

Currently, imaging equipment such as CT and MRI primarily identify abnormal lymph nodes by visually assessing their size, shape, and contrast-enhanced regions. PET-CT can identify LNM (e.g., short axis diameter of lymph node > 10 mm on CT, maximum standardized uptake value ≥ 2.5 on PET/CT) through the uptake of abnormal radioactive elements. However, the involvement of too many subjective factors in evaluation can easily lead to a bias in the diagnostic results (34, 35). Although widely validated, existing mainstream nomogram models have yet to demonstrate significant predictive performance. Bandini et al., Hueting et al., Oderda et al., and Gandaglia et al. each validated different nomogram models using large datasets, revealing variable predictive accuracy (8–10, 12). The variances might arise from the discrepant composition of patients in the validation datasets and the circumstance that they originate from different regions. Di et al. reported that among high-risk prostate PCa patients, all four evaluated models systematically overestimated the risk of LNM to varying degrees (36). Specifically, the MSKCC, Briganti 2012, Briganti 2017, and Briganti 2019 nomograms exhibited similar predictive performance for LNM, with respective AUC values of 0.526, 0.548, 0.555, and 0.573. Moreover, these nomograms displayed relatively high sensitivity (0.973, 0.991, 0.973, and 0.959, respectively) yet exhibited extremely low specificity (0.078, 0.093, 0.140, and 0.183, respectively). These results suggest that there is scope for improvement in the existing models in terms of accurately predicting LNM.

**Figure 10:**
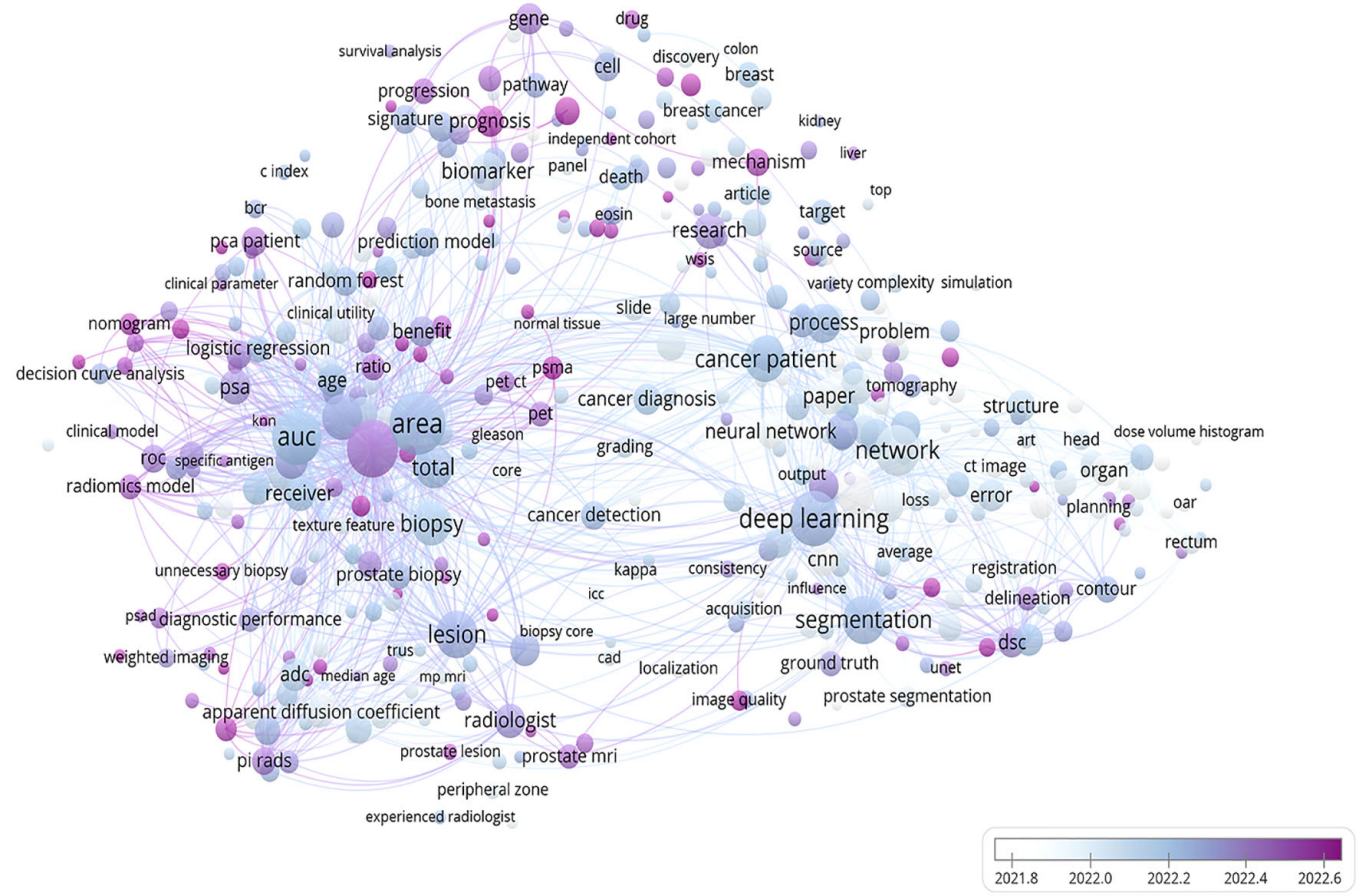
A bibliometric network map of the application of radiomics technology in prostate cancer. This figure depicts the findings of a recent 5-year study on prostate cancer utilizing radiomics technology. The bar graph in the lower right corner illustrates the transition from white to purple, symbolizing the historical progression to the present. Each circle represents a specific theme or keyword, with its size corresponding to publication frequency. The figure was created using VOS viewer (version 1.6.20, www.vosviewer.com) based on scientific articles in the Web of Science database.

Image-based radiomics techniques partially address this limitation by enabling quantitative feature analysis for assessing disease progression. A bibliometric analysis of recent publications on PCa and radiomics technology within the Web of Science database over the last five years reveals a close association between PCa and radiomics as well as key concepts such as AUC, deep learning, and biomarkers (**Figure 10**). Numerous studies have now successfully constructed predictive models for predicting LNM in PCa based on extracted imaging features from CT scans, MRIs, or PET-CT scans (37). However, there is currently a lack of robust evidence supporting the ability of radiomics technology to diagnose diseases, and there are fewer systematic reviews and meta-analyses on the application of radiomics in PCa LNM.

In this study, radiomics technology demonstrates well sensitivity and specificity in predicting LNM. Our findings align with those of Abbaspour et al., who conducted a meta-analysis of 36 studies on the diagnostic performance of radiomics technology for predicting LNM of colorectal cancer, yielding a combined AUC, sensitivity, and specificity of 0.814 ([95% CI: 0.78 - 0.85]), 0.77 ([95% CI: 0.69 - 0.84]), and 0.73 ([95% CI: 0.67 - 0.78]), respectively (38). Li et al. included 12 studies in their analysis to evaluate the diagnostic ability of radiomics in predicting cervical cancer LNM, with a combined AUC, sensitivity, and specificity of 0.83 ([95% CI: 0.76 - 0.89]), 0.80 ([95% CI: 0.72 - 0.87]), and 0.76 ([95% CI: 0.72 - 0.80]), respectively (39). While our findings demonstrate superior diagnostic performance compared to the two previous studies, it does not imply that radiomics technology is more effective in predicting LNM of PCa than in predicting metastasis of other tumors. Such differences may reflect heterogeneity among pathological tumor types, variability in imaging equipment and features, and challenges in reproducing modeling methodologies (23).

In terms of clinical practicality, Fagan nomogram analysis shows that the model based on radiomics features can increase the post-test probability of positive results to 62% and reduce the post-test probability of negative results to 8%. Compared with the widely used MSKCC nomogram and Briganti nomogram (2012, 2017, 2019 editions), the radiomics model demonstrates higher diagnostic efficacy (with a better AUC value), and due to its non-invasive advantage, it may reduce patients’ reliance on prostate biopsy and thereby avoid related complications. However, the current negative posterior probability (8%) of the radiomics model is still higher than that of MSKCC (5%) and Briganti nomogram (minimum 2%), its false negative risk limits the reliability of its sole clinical application. Therefore, at this stage, the radiomics model cannot completely replace the MSKCC or Briganti nomogram, but can serve as one of the supplementary tools for clinical decision-making.

During the process of constructing predictive models, owing to the high dimensionality of radiomics features, the RF algorithm exhibits superiority in handling complex nonlinear relationships, whereas LASSO algorithm is more proficient in fitting linear relationships (23). Nevertheless, in this study, the combined sensitivity of LASSO algorithm was 0.96 ([95% CI: 0.90 - 1.00]) (*p* = 0.02), while that of RF was 0.48 ([95% CI: 0.16 - 0.80]) (*p* = 0.01). Despite the statistical significance of this difference, substantial variations in training and validation set sizes, data distributions, and hyperparameter optimization strategies across studies may have influenced model generalizability. For example, in the studies by Cysouw et.al, Hou et.al, and Luning et.al, the sample sizes of the validation sets of the RF models (14, 50, and 51, respectively) were markedly smaller than those of the LASSO models in the studies by Liu et al. and Peeken et al. (208 and 102, respectively). Furthermore, in small-sample studies, the RF algorithm is more susceptible to overfitting, resulting in the degradation of the performance of the validation set, while the regularization property of LASSO enables it to perform more stably under small-sample conditions (40). During the hyperparameter optimization process, RF algorithm is more sensitive to hyperparameters (such as tree depth and node size, etc.). If the tuning is inadequate or the data is limited, its performance may be underestimated. Although the current results indicate that LASSO algorithm may be more robust, the evidential strength of this conclusion is limited due to the heterogeneity of the datasets and methodological differences. Currently, there is no optimal modeling approach in model construction, and distinct modeling methods possess obvious inherent limitations, such as the assumption of feature independence in Logit models, the requirement for feature discretization in Bayesian networks, and the dependence on network configuration in DL. In the future, while pursuing the predictive ability of the models, it is of greater significance to ensure that the developed models are fully reproducible (23, 41).

The validation methods for the model include internal validation and external validation. At present, for the developed radiomics model, most are evaluated by internal validation to assess the predictive performance of the model. According to the literature, external validation is recommended for datasets with more than 50 samples, while resampling methods are preferable for smaller datasets (42). Among various resampling methods, cross-validation and bootstrap methods are the most widely used. Cross-validation mainly focuses on evaluating the predictive performance of the model (calculating some statistical data on the missing samples and evaluating the model’s predictability) (43). Bootstrap method primarily focuses on the statistical evaluation of the model, rather than assessing the predictive validity of the samples missed in each iteration (44–46). Our findings show that bootstrap method can improve model sensitivity (0.97 vs. 0.72) compared to cross-validation. However, it does not imply that the bootstrap method is the best way to validate models. Model performance on new data largely depends on the quality of the training data. Furthermore, the best model is not the one that is best suited for calibrating the data or providing the best results in validation, but rather the one that can predict new samples with high reliability and stable results (47).

ICC is a statistical method based on analysis of variance, commonly used to analyze continuous numerical data with a range of ratio indicators between 0 and 1 (48).

In radiomics model construction, ICC assesses the reproducibility and robustness of imaging features extracted from tumor lesions across different individuals, segmentation methods, and time points. This assessment encompasses inter-observer or intra-observer heterogeneity. Previous reviews have indicated that most imaging features demonstrate high robustness to inter-observer or intra-observer heterogeneity, regardless of the ICC threshold set and the type of tumor (49). In line with the findings of the appeal, our study suggests that unprocessed imaging features may result in a reduction in diagnostic specificity, yielding a combined specificity of 0.73 ([95% CI: 0.53 - 0.92]) (p=0.04). Nevertheless, the threshold setting for ICC value has not been standardized yet, and there exist numerous sources of heterogeneity including imaging scanner parameters, imaging resolution, tumor segmentation methods, feature extraction software, etc., thus far precluding any quantitative evaluation of ICC (50).

Among the 10 studies included in this analysis, the RQS was 16.5, which is below both the 50% threshold (18/36) and the 60% threshold (21.6/36) of the maximum score. Nevertheless, it is noteworthy that the average RQS score surpassed those reported in other studies (38, 51). Subgroup analysis revealed that the diagnostic specificity for RQS score ≥17 was 0.77 ([95% CI: 0.65 - 0.88]) (p=0.01), with a decrease in specificity as the score increased, possibly attributed to inter-rater variability in the RQS scoring process and challenges associated with replicating the RQS score (52). Furthermore, it is important to note that currently, the applicability of the RQS tool is limited to traditional radiomics workflows, lacking corresponding evaluation criteria for DL research. Studies on identifying the status of LNM using DL algorithms are constantly increasing (53–55). However, this approach to data processing differs from the classic feature processing, selection, and model tuning procedures in radiomics. For instance, traditional radiomics features are based on manual segmentation, while DL algorithms extract features directly from images and have a black-box nature, making them not directly applicable to the “feature reproducibility” and “feature interpretability” scoring items in RQS tool. Additionally, DL algorithms mainly enhance data reliability through methods such as image rotation and flipping, but there are no corresponding evaluation criteria for these methods in the RQS tool. Therefore, the RQS tool still needs further improvement to adapt to the progress of algorithms.

This study has several limitations. First, the number of articles meeting the inclusion criteria is limited, and some data are derived through calculations. Second, most included studies were retrospective, with relatively few prospective studies, potentially weakening the strength of evidence, potentially leading to a decline in the strength of evidence. Third, while the number of studies on predictive models based on radiomics features has increased annually, there are very few multi-center or cross-regional external validation studies on the developed models, which may undermine the reproducibility and credibility of these models. Fourth, imaging features may be influenced by factors such as imaging equipment instrument protocols, contrast agent types, tumor segmentation methods, feature extraction software, and modeling methods.

Such variability may contribute to differences in diagnostic performance and underscores the need for authoritative, standardized operational guidelines. Finally, there is currently no fully unified expert consensus on quality assessment procedures for imaging genomics operations despite widespread use of RQS as a quality assessment tool; further improvements are necessary.

## 5 Conclusions

The promising diagnostic capability of radiomics technology in preoperatively predicting LNM in PCa holds potential clinical relevance for guiding treatment decisions. Nevertheless, the current limitations associated with this technology may restrict its immediate clinical applicability. Further comprehensive research is warranted to validate the findings of this study and facilitate the integration of this technology into clinical practice.

## Data Availability

The original contributions presented in the study are included in the article/**Supplementary Material**. Further inquiries can be directed to the corresponding author.
