# Supplementary material for: Application of radiomics-based prediction model to predict preoperative lymph node metastasis in prostate cancer: a systematic review and meta-analysis
Source: Front Oncol. 2025 Jun 20;15:1577794. doi: 10.3389/fonc.2025.1577794 (PMC12226473; doi:10.3389/fonc.2025.1577794)

**Supplement materials**

**S1.** The detailed Search strategy of the databases.

**Pubmed**

(("Artificial Intelligence"[Mesh] OR "Artificial intelligence" OR "deep learning" OR "convolutional neural network" OR "machine learning" OR "automatic detection" OR "radiomics" OR "radiomic") AND ("Magnetic Resonance Imaging"[Mesh] OR "Tomography, X-Ray Computed"[Mesh] OR "CT" OR "MRI") AND ("Lymphatic Metastasis"[Mesh] OR "Lymph node" OR "lymph node metastasis" OR "LNM")) AND ((Neoplasms, Prostatic OR Neoplasm, Prostatic OR Prostatic Neoplasm OR Prostate Neoplasms OR Neoplasms, Prostate OR Neoplasm, Prostate OR Prostate Neoplasm OR Prostate Cancer OR Cancer, Prostate OR Cancers, Prostate OR Prostate Cancers OR Cancer of Prostate OR Cancer of the Prostate OR Prostatic Cancer OR Cancer, Prostatic OR Cancers, Prostatic OR Prostatic Cancers) OR ("Prostatic Neoplasms"[Mesh]))

**Web of Science**

#1.TS=(Prostatic Neoplasm OR Prostate Neoplasms OR Prostate Neoplasm OR Prostate Cancer OR Prostate Cancers OR Cancer of Prostate OR Cancer of the Prostate OR Prostatic Cancer OR Prostatic Cancers)

#2.TS=(Artificial intelligence OR deep learning OR convolutional neural network OR machine learning OR automatic detection OR radiomics OR radiomic)

#3.TS=(Magnetic Resonance Imaging OR MRI)

#4.TS=(Tomography, X-Ray Computed OR CT)

#5.TS=(Lymphatic Metastasis OR Lymph node OR lymph node metastasis OR LNM)

Combined #1 AND #2 AND #3 AND #5

**Embase**

#1'Neoplasms, Prostatic':ab,kw,ti OR 'Neoplasm, Prostatic':ab,kw,ti OR 'Prostatic Neoplasm':ab,kw,ti OR 'Prostate Neoplasms':ab,kw,ti OR 'Neoplasms, Prostate':ab,kw,ti OR 'Neoplasm, Prostate':ab,kw,ti OR 'Prostate Neoplasm':ab,kw,ti OR 'Prostate Cancer':ab,kw,ti OR 'Cancer, Prostate':ab,kw,ti OR 'Cancers, Prostate':ab,kw,ti OR 'Prostate Cancers':ab,kw,ti OR 'Cancer of Prostate':ab,kw,ti OR 'Cancer of the Prostate':ab,kw,ti OR 'Prostatic Cancer':ab,kw,ti OR 'Cancer, Prostatic':ab,kw,ti OR 'Cancers, Prostatic':ab,kw,ti OR 'Prostatic Cancers':ab,kw,ti

#2'Artificial intelligence':ab,kw,ti OR 'deep learning':ab,kw,ti OR 'convolutional neural network':ab,kw,ti OR 'machine learning':ab,kw,ti OR 'automatic detection':ab,kw,ti OR 'radiomics':ab,kw,ti OR 'radiomic':ab,kw,ti

#3'Magnetic Resonance Imaging':ab,kw,ti OR 'MRI':ab,kw,ti

#4'Tomography, X-Ray Computed':ab,kw,ti OR 'CT':ab,kw,ti

#5'Lymphatic Metastasis':ab,kw,ti OR 'Lymph node':ab,kw,ti OR 'lymph node metastasis':ab,kw,ti OR 'LNM':ab,kw,ti

Combined #1 AND #2 AND #3 AND #5

**Cochrane Library**

#1 (Artificial intelligence):ti,ab,kw OR (deep learning):ti,ab,kw OR (convolutional neural network):ti,ab,kw OR (machine learning):ti,ab,kw OR (automatic detection):ti,ab,kw OR (radiomics):ti,ab,kw) OR (radiomic):ti,ab,kw

#2 (Prostatic Neoplasms) OR (Neoplasms, Prostatic):ti,ab,kw OR (Neoplasm, Prostatic):ti,ab,kw OR (Prostatic Neoplasm):ti,ab,kw OR (Prostate Neoplasms):ti,ab,kw OR (Neoplasms, Prostate):ti,ab,kw OR (Neoplasm, Prostate):ti,ab,kw OR (Prostate Neoplasm):ti,ab,kw OR (Prostate Cancer):ti,ab,kw OR (Cancer, Prostate):ti,ab,kw OR (Cancers, Prostate):ti,ab,kw OR (Prostate Cancers):ti,ab,kw OR (Cancer of Prostate):ti,ab,kw OR (Cancer of the Prostate):ti,ab,kw OR (Prostatic Cancer):ti,ab,kw OR (Cancer, Prostatic):ti,ab,kw OR (Cancers, Prostatic):ti,ab,kw OR (Prostatic Cancers):ti,ab,kw) #3 (Magnetic Resonance Imaging:ti,ab,kw OR (MRI):ti,ab,kw

#4 (Tomography, X-Ray Computed) OR (CT):ti,ab,kw

#5 (Lymphatic Metastasis) OR (Lymph node):ti,ab,kw OR (lymph node metastasis):ti,ab,kw OR (LNM):ti,ab,kw

Combined #1 AND #2 AND #3 AND #5

**Sinomed**

"Artificial Intelligence" AND ("Magnetic Resonance Imaging" OR "Tomography, X-Ray Computed" OR "CT" OR "MRI") AND "Lymph node" AND "Prostatic Neoplasms"

**S2.** Quality assessment of each included study based on the Quality Assessment of Diagnostic Accuracy Studies-2 (QUADAS-2)

|  | **Source** | **RISK OF BIAS** | | | | | | | | **APPLICABILITY CONCERNS** | | |
| --- | --- | --- | --- | --- | --- | --- | --- | --- | --- | --- | --- | --- |
|  |  | **PATIENT SELECTION** | | | **INDEX TEST** | | | **REFERENCE STANDARD** | **FLOW AND TIMING** | **PATIENT SELECTION** | **INDEX TEST** | **REFERENCE STANDARD** |
|  |  | Was the statistical management adequate? | Were the inclusion/exclusion criteria specified? | Was the type of study (retrospective or prospective) specified? | Were the imaging acquisition protocol and the segmentation method(s) detailed? | Was the image  processing approach detailed? | Was the validation independent (i.e., no internal)? | Was the reference  standard adequate? | Was there an  appropriate interval between index test  and reference standard? |  |  |  |
| 1 | Bourbonne 2021 | yes | yes | yes | yes | yes | no | yes | unclear | yes | yes | yes |
| 2 | Cysouw 2021 | yes | yes | yes | yes | yes | no | yes | unclear | yes | yes | yes |
| 3 | Hou 2021 | yes | yes | yes | yes | yes | yes | yes | unclear | yes | yes | yes |
| 4 | Lai 2021 | yes | yes | yes | yes | yes | no | yes | unclear | yes | yes | yes |
| 5 | Liu 2022 | yes | yes | yes | yes | yes | no | yes | unclear | yes | yes | yes |
| 6 | Liu-2 2022 | yes | yes | yes | yes | yes | no | yes | unclear | yes | yes | yes |
| 7 | Liu-3 2022 | yes | yes | yes | yes | yes | no | yes | unclear | yes | yes | yes |
| 8 | Luining 2023 | yes | yes | yes | yes | yes | yes | yes | unclear | yes | yes | yes |
| 9 | Peeken 2021 | yes | yes | yes | yes | yes | yes | yes | unclear | yes | yes | yes |
| 10 | Zamboglou 2019 | yes | yes | yes | yes | yes | no | yes | unclear | yes | yes | yes |
| 11 | Zheng 2022 | yes | yes | yes | yes | yes | no | yes | unclear | yes | yes | yes |

**S3.1** The Radiomics Quality Score in six domains.

| **Domain** | **No.** | **RQS scoring item** | **Points and Interpretation** |
| --- | --- | --- | --- |
| **Domain 1: Protocol quality and stability in image and segmentation (0 to 5)** | **1** | **Image protocol quality** - well-documented image protocols (for example, contrast, slice thickness, energy, etc.) and/or usage of public image protocols allow reproducibility/replicability | + 1 if protocols are well-documented  + 1 if public protocol is used |
|  | **2** | **Multiple segmentations** - possible actions are: segmentation by different physicians/algorithms/software, perturbing segmentations by (random) noise, segmentation at different breathing cycles. Analyse feature robustness to segmentation variabilities | + 1 if segmented multiple times (different physicians, algorithms, or perturbation of regions of interest) |
|  | **3** | **Phantom study on all scanners** - detect inter-scanner differences and vendor-dependent features. Analyse feature robustness to these sources of variability | + 1 if texture phantoms were used for feature robustness assessment |
|  | **4** | **Imaging at multiple time points** - collect images of individuals at additional time points. Analyse feature robustness to temporal variabilities (for example, organ movement, organ expansion/ shrinkage) | + 1 multiple time points for feature robustness assessment |
| **Domain 2: Feature selection and validation (- 8 to 8)** | **5** | **Feature reduction or adjustment for multiple testing** - decreases the risk of overfitting. Overfitting is inevitable if the number of features exceeds the number of samples. Consider feature robustness when selecting features | - 3 if neither measure is implemented  + 3 if either measure is implemented |
|  | **12** | **Validation** - the validation is performed without retraining and without adaptation of the cut-off value, provides crucial information with regard to credible clinical performance | − 5 if validation is missing  + 2 if validation is based on a dataset from the same institute/  + 3 if validation is based on a dataset from another institute/  + 4 if validation is based on two datasets from two distinct institutes/  +4 if the study validates a previously published signature/  +5 if validation is based on three or more datasets from distinct institutes  *Datasets should be of comparable size and should have at least 10 events per model feature |
| **Domain 3: Biologic/clinical validation and utility (0 to 6)** | **6** | **Multivariable analysis with non-radiomics features** (for example, EGFR mutation) - is expected to provide a more holistic model. Permits correlating/inferencing between radiomics and non-radiomics features | + 1 if multivariable analysis with non-radiomics features |
|  | **7** | **Detect and discuss biological correlates** - demonstration of phenotypic differences (possibly associated with underlying gene–protein expression patterns) deepens understanding of radiomics and biology | + 1 if present |
|  | **13** | **Comparison to ‘gold standard**’ - assess the extent to which the model agrees with/is superior to the current ‘gold standard’ method (for example, TNM-staging for survival prediction). This comparison shows the added value of radiomics | + 2 for comparison to gold standard |
|  | **14** | **Potential clinical utility** - report on the current and potential application of the model in a clinical setting (for example, decision curve analysis) | + 2 for reporting potential clinical utility |
| **Domain 4:**  **Model performance index (0 to 5)** | **8** | **Cut-off analyses** - determine risk groups by either the median, a previously published cut-off or report a continuous risk variable. Reduces the risk of reporting overly optimistic results | + 1 if cutoff either pre-defined or at median or continuous risk variable reported |
|  | **9** | **Discrimination statistics** - report discrimination statistics (for example, C-statistic, ROC curve, AUC) and their statistical significance (for example, p-values, confidence intervals). One can also apply resampling method (for example, bootstrapping, cross-validation) | + 1 if a discrimination statistic and its statistical significance are reported  + 1 if a resampling method technique is also applied |
|  | **10** | **Calibration statistics** - report calibration statistics (for example, Calibration-in-the-large/slope, calibration plots) and their statistical significance (for example, *P*-values, confidence intervals). One can also apply resampling method (for example, bootstrapping, cross-validation) | + 1 if a calibration statistic and its statistical significance are reported  + 1 if a resampling method technique is also applied |
| **Domain 5:**  **High level of evidence (0 to 8)** | **11** | **Prospective study registered in a trial database** - provides the highest level of evidence supporting the clinical validity and usefulness of the radiomics biomarker | + 7 for prospective validation of a radiomics signature in an appropriate trial |
|  | **15** | **Cost-effectiveness analysis** - report on the cost-effectiveness of the clinical application (for example, QALYs generated) | + 1 for cost-effectiveness analysis |
| **Domain 6:**  **Open science and data (0 to 4)** | **16** | **Open science and data** - make code and data publicly available. Open science facilitates knowledge transfer and reproducibility of the study | + 1 if scans are open source  + 1 if region of interest segmentations are open source  + 1 if code is open source  + 1 if radiomics features are calculated on a set of representative ROIs and the calculated features and representative ROIs are open source |
| **Total points (36 = 100%)** | | | |

**S3.2** The Radiomics Quality Score of the included studies in six domains.

| Author/year | Domain (Score) | | | | | | | | | | | | | | | | Total scores |
| --- | --- | --- | --- | --- | --- | --- | --- | --- | --- | --- | --- | --- | --- | --- | --- | --- | --- |
|  | Domain 1 | | | | Domain 2 | | Domain 3 | | | | Domain 4 | | | Domain 5 | | Domain 6 |  |
|  | Image protocol quality | Multiple segmentations | Phantom study on all scanners | Imaging at multiple time points | Feature reduction or adjustment for multiple testing | Validation | Multivariable analysis with non-radiomics features | Detect and discuss biological correlates | Comparison to ‘gold standard’ | Potential clinical utility | Cut-off analyses | Discrimination statistics | Calibration statistics | Prospective study registered in a trial database | Cost-effectiveness analysis | Open science and data |  |
| Bourbonne 2021 | 1 | 0 | 0 | 0 | 3 | 2 | 1 | 0 | 2 | 2 | 1 | 2 | 2 | 0 | 1 | 2 | 19 |
| Cysouw 2021 | 1 | 1 | 0 | 0 | 3 | 2 | 1 | 0 | 2 | 0 | 0 | 2 | 0 | 7 | 0 | 2 | 21 |
| Hou 2021 | 1 | 1 | 0 | 0 | 3 | 4 | 1 | 0 | 2 | 0 | 0 | 2 | 0 | 0 | 0 | 1 | 15 |
| Lai 2021 | 1 | 1 | 0 | 0 | 3 | 2 | 0 | 0 | 2 | 0 | 0 | 2 | 0 | 0 | 0 | 2 | 13 |
| Liu 2022 | 1 | 1 | 0 | 1 | 3 | 2 | 0 | 0 | 2 | 2 | 1 | 2 | 0 | 0 | 1 | 2 | 18 |
| Liu-2 2022 | 1 | 0 | 0 | 0 | 3 | 4 | 0 | 0 | 2 | 2 | 1 | 2 | 2 | 0 | 1 | 1 | 19 |
| Liu-3 2022 | 1 | 1 | 1 | 0 | 3 | 2 | 1 | 0 | 2 | 2 | 0 | 2 | 2 | 0 | 1 | 1 | 19 |
| Luining 2023 | 1 | 0 | 0 | 0 | 3 | 4 | 1 | 0 | 2 | 0 | 0 | 2 | 0 | 0 | 0 | 1 | 14 |
| Peeken 2021 | 1 | 1 | 0 | 0 | 3 | 2 | 1 | 0 | 2 | 2 | 0 | 2 | 2 | 0 | 1 | 2 | 19 |
| Zamboglou 2019 | 1 | 1 | 0 | 0 | 3 | 2 | 1 | 0 | 2 | 0 | 0 | 2 | 0 | 0 | 0 | 1 | 13 |
| Zheng 2022 | 1 | 1 | 0 | 0 | 3 | 2 | 1 | 0 | 2 | 0 | 0 | 2 | 0 | 0 | 0 | 1 | 13 |

**S4.** The calculation formulas of corresponding indicators and the operation manual of R package.

| **Measure** | **Formula*** |
| --- | --- |
| Sensitivity | $\frac{TP}{P} =\frac{TP}{TP+FN}$ |
| Specificity | $\frac{TN}{N}= \frac{TN}{TN+FP}$ |
| Accuracy | $\frac{TP+TN}{P+N}= \frac{TP+TN}{TP+TN+FP+FN}$ |
| PPV | $\frac{TP}{TP+FP}$ |
| NPV | $\frac{TN}{TN+FN}$ |
| SE | $\frac{\left( Upper Limit-Lower Limit \right)}{2t}$ ; t=TINV (1-0.95, N-1) |
| 95% Confidence Interval | $best estimate+/-\left( 1.96 \right)*(SE)$ |
| PLR | $\frac{TP/(TP+TN)}{FP/+FP+FN}$ |
| NLR | $\frac{FN/(TP+FN)}{TN/(FP+TN)}$ |
| DOR | $\frac{TP/FN}{FP/TN}$ |

*The meta-analysis and data calculation based on the R Studio was elaborated in the reference handbook^[1]^ (https://bookdown.org/MathiasHarrer/Doing_Meta_Analysis_in_R/).

[1] HARRER M, CUIJPERS P, FURUKAWA T A, EBERT D D. Doing meta-analysis with r: A hands-on guide [M]. 1st ed. Boca Raton, FL and London: Chapman & Hall/CRC Press, 2021.

**S5.** The figure of positive likelihood ratio and negative likelihood ratio.

**S6.** The figure of diagnostic scores and odds ratios.

**S7.** The figure of subgroup analysis results with discrepancy significance in the included study.


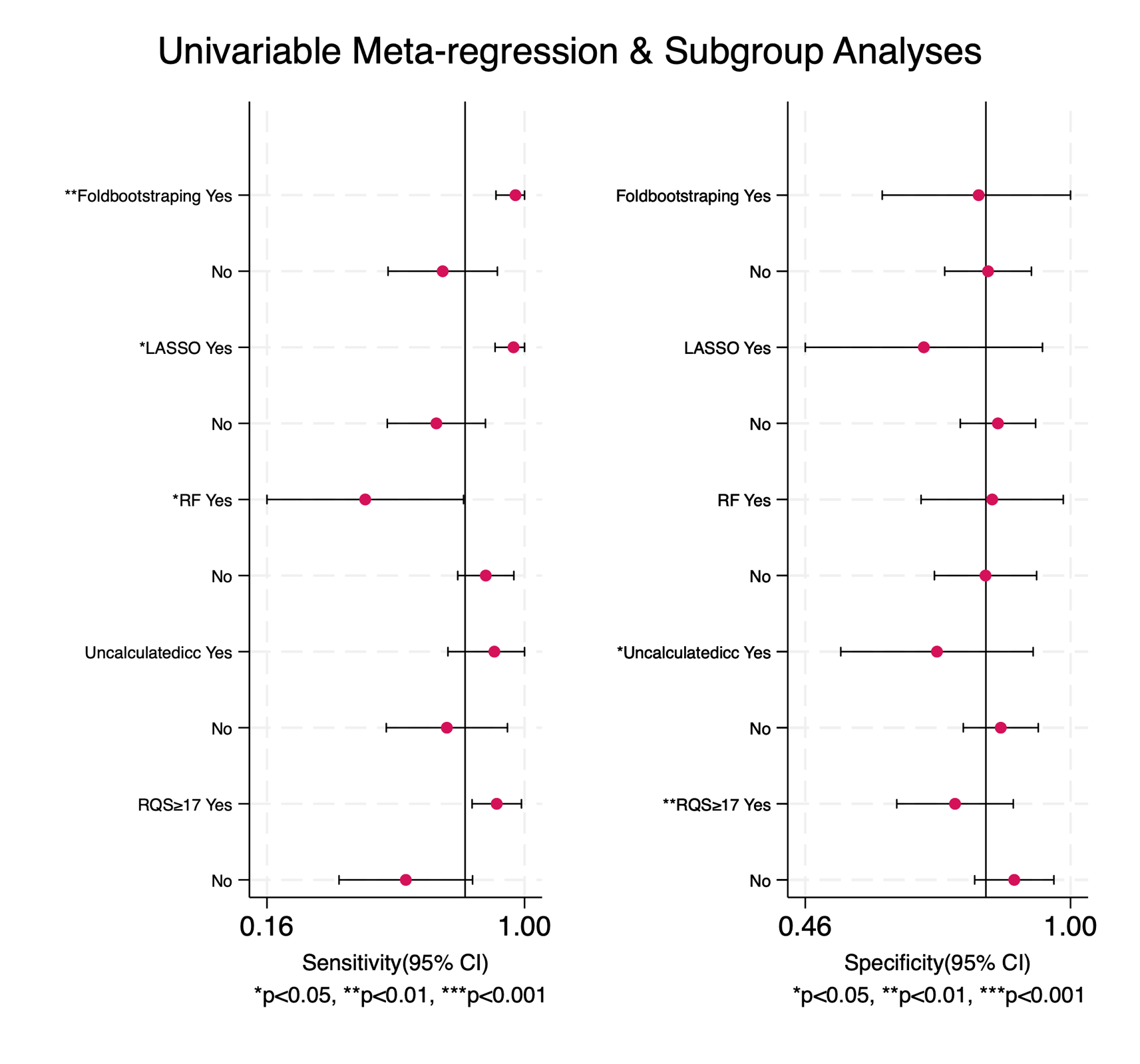


**S8.** The figure of subgroup analysis in pooled AUC.


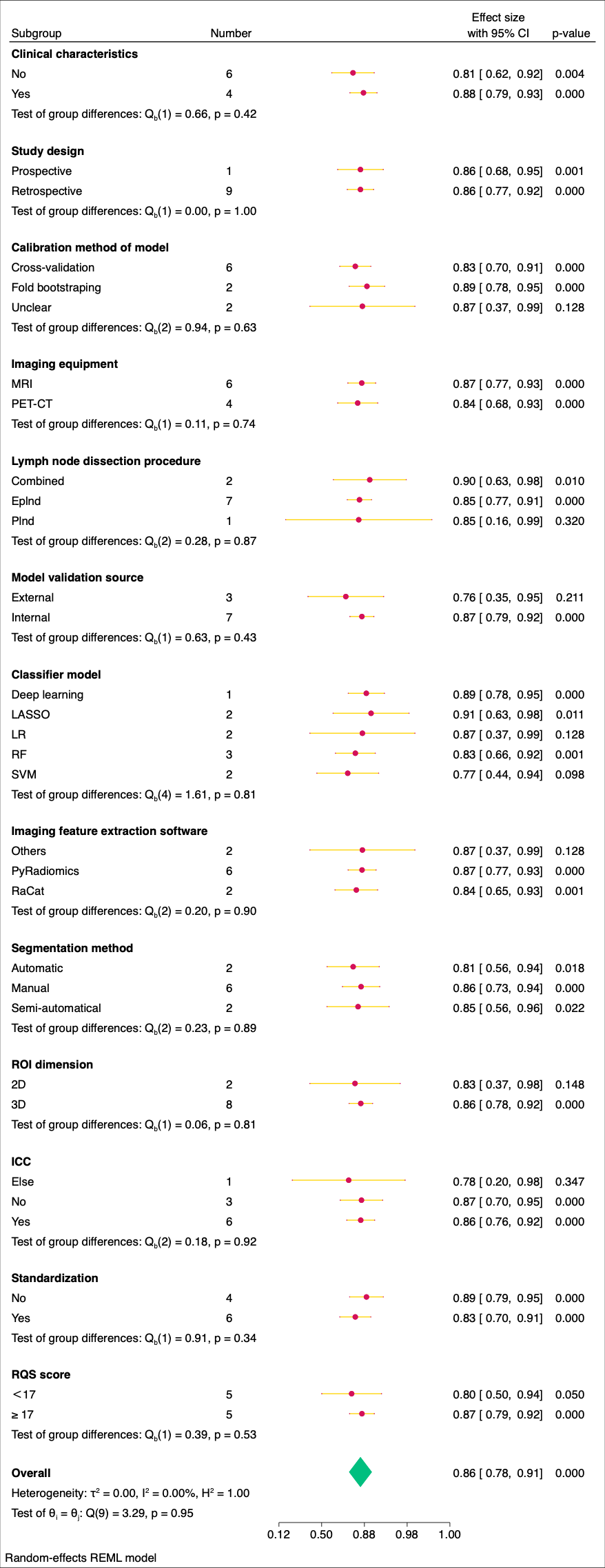

Supplement: Supplementary file 1 [file DataSheet1.docx]
